# Genomic Characterization of Chicken Anemia Virus in Broilers in Shandong Province, China, 2020–2021

**DOI:** 10.3389/fvets.2022.816860

**Published:** 2022-03-17

**Authors:** Ling Liu, Yuyan Li, Mingrong Yin, Peng Zhao, Longzong Guo, Yixin Wang

**Affiliations:** ^1^College of Animal Science and Veterinary Medicine, Shandong Agricultural University, Tai'an, China; ^2^Shandong Provincial Key Laboratory of Animal Biotechnology and Disease Control and Prevention, Tai'an, China; ^3^Shandong Yisheng Livestock and Poultry Breeding Co., Ltd., Yantai, China

**Keywords:** chicken anemia virus, full-length genome, molecular characterization, phylogenetic analysis, recombination analysis

## Abstract

Chicken infectious anemia (CIA), caused by chicken anemia virus (CAV), is an immunosuppressive disease characterized by growth retardation, aplastic anemia, lymphoid depletion, and immunodepression in young chickens. In this study, 33 CAV strains were isolated from broilers in Shandong Province during 2020–2021. Phylogenetic analysis of full-length genome sequences showed that most CAV strains isolated in this study were scattered across different branches, but mainly clustered in two genotypes, indicating a certain regional characteristic. Analysis of VP1 protein identified several amino acid substitutions which were relevant with the virulence and virus spread efficiency. Interestingly, four putative DNA recombination events were detected in the genomes of novel isolated CAV strains. In summary, this study demonstrated a genomic diversity of CAV in broilers isolated in Shandong Province during 2020–2021, and provided information for the further study of CAV molecular epidemiology and viral evolution.

## Introduction

Chicken infectious anemia (CIA) is an immunosuppressive disease caused by chicken anemia virus (CAV), which is mainly characterized by aplastic anemia, growth retardation, lymphoid tissue atrophy, and immunosuppression (1). It has brought huge economic losses to the global poultry industry. Chicken is the main natural host of CAV, and all kinds of chicken breeds are susceptible to this virus. CAV mainly infects 10–14 days-old chickens, resulting in anemia, thymus atrophy and yellow bone marrow. Although CAV only induces a mild subclinical infection in adult chickens, infected chickens are usually manifested with immunosuppression and become more sensitive to the secondary viral, bacterial or fungal infection (2).

CAV, belonging to the *Circoviridae*, the genus *Gyrovirus*, family *Anelloviridae*, is a small DNA virus with an icosahedral capsid about 20 nm diameter in size (3). The CAV genome, which consists of 2,298 or 2,319 nucleotides, contains three overlapping open reading frames (ORFs), which are translated into three main viral proteins, namely capsid protein VP1 (51.6 kDa), associated protein VP2 (24 kDa), and apoptin protein VP3 (13.6 kDa) (4). VP1 is the major viral structural protein of CAV, which contains abundant neutralization antigenic epitope and plays a key role in virus growth and transmission (5). VP2 is a non-structural protein with a phosphatase activity, which is related to viral assembly in the infectious cycle. VP2 is considered as a scaffold protein assisting VP1 protein folding du ring viral particle assembly. In addition, co-expression of VP1 and VP2 can induce neutralizing antibody in the host cell (6, 7). VP3, also known as apoptin, is a mainly virulence factor of the virus inducing extensive lymphoatrophy and anemia in infected chickens. It can trigger apoptosis in host cells and several tumor cell lines independent of p53 activation (8, 9).

Since its first report in 1979 in Japan (10), CAV had been spread in many countries worldwide (11–16). To date, all CAV isolates had a similar antigenic characteristic and belong to the same serotype, while the genomic sequence and virulence between different strains were divergent. CAV was first isolated and reported in China in 1992, and since then, the positive rate of CAV infection had been increasing in chicken farms in different provinces in China, posing a serious threat to the production of poultry industry. In recent years, epidemiological investigations and viral genome analysis of CAV had been conducted by researchers (17–21). According to the epidemiological survey data, CAV had been widely existed in various types of chicken breeds in China, including laying hens, broilers, and Chinese indigenous chickens. More importantly, CAV genomic fragments could even be detected in the feces of some mammals, suggesting a possibility transmission route of CAV by mammals like cats and dogs (18).

Shandong Province is one of the largest poultry breeding areas in China, and the breeding stock of progenitor broilers approximately accounts for a quarter of China. In order to investigate the CAV infection status recent years in broilers in Shandong province, samples of broilers suspected to be infected with CAV were collected from January 2020 to October 2021. Subsequently, positive samples were homogenized and inoculated into MDCC-MSB1 cells, respectively, and 33 CAV strains were successfully isolated. Finally, the full-length genomic sequences of these 33 strains were analyzed. Our findings may reveal the molecular epidemiological characteristics of CAV strains in Shandong Province and contribute to the understanding of CAV viral evolution in broilers.

## Materials and Methods

### Clinical Samples

Clinical samples including bone marrow, liver, spleen, and thymus were collected from diseased birds during 2020–2021. For progenitor and parent broilers older than 7 weeks, CAV infection would be suspected especially when thymic atrophy or yellow bone marrow occurred during autopsy. For commercial broilers or backyard broilers, CAV infection would be suspected when chickens manifested with depression, emaciation, growth retardation or immunosuppression at the early age. A total of 73 clinical samples were collected and details of those 73 samples was shown in Supplementary Table 1. All samples were stored at −80°C.

### Viral DNA Detection by PCR

DNA of each clinical samples were extracted using TIANamp Genomic DNA Kit (Tiangen Biotechnology Co., Ltd., China) following the manufacturer's instructions and then detected by PCR. The sequence of primers was designed based on the Cux-1 sequence (accession NO. M55918) and shown in Table 1. PCR was performed in a 25 μl reaction volume containing 0.5 μl forward primers and downstream primers (10 mM), 1 μl DNA, 2.5 μl buffer, 2 μl dNTPs (2.5 mM), 0.5 μl high fidelity DNA polymerase (TaKaRa Bio, Inc., Dalian, China), and 18 μl deionized water. The PCR reaction conditions were as following: 98°C pre-denaturation for 1 min, followed by 98°C denaturation for 10 s, 56°C annealing for 1 min, and 72°C extension for 30 s; The extension was carried out for another 10 min before terminating the reaction at 4°C. PCR products were analyzed by 1% agarose gel electrophoresis.

**Table 1:** Sequences of primers used in this study.

**Primers**	**The sequences of the primers (5′ → 3′)**	**Sizes**
CAV-F	GCATTCCGAGTGGTTACTATTCC	982 bp
CAV-R	CGTCTTGCCATCTTACAGTCTTAT	
CAV-com-F1	GCATTCCGAGTGGTTACTATTCC	842 bp
CAV-com-R1	CGTCTTGCCATCTTACAGTCTTAT	
CAV-com-F2	CGAGTACAGGGTAAGCGAGCTAAA	990 bp
CAV-com-R2	TGCTATTCATGCAGCGGACTT	
CAV-com-F3	ACGAGCAACAGTACCCTGCTAT	802 bp
CAV-com-R3	CTGTACATGCTCCACTCGTT	

### Virus Isolation

Positive samples were used for virus isolation by inoculating MDCC-MSB1 cells (22). Briefly, ~0.5 g positive samples were homogenized with 2 mL PBS buffer and centrifuged at 12,000 rpm at 4°C, respectively. The supernatant was filtered by 0.22 μM filter, and MDCC-MSB1 cells in 25 cm^2^ culture bottle were infected with 0.5 mL filtered solution, followed by incubation at 37°C for 1 h. Then, the supernatant was removed by centrifugation, 5 ml RPMI 1640 medium was added again to resuspend the cells, and cultured for another 3 days at 37°C. The cells were continuously passaged until obvious cytopathy appeared. The cellular supernatant was collected and the presence of viral DNA was detected by PCR as previously mentioned.

### Genomic Sequencing of CAV Isolates

According to the published genomic sequences of CAV reference strains in the GenBank, four pairs of primers were designed and synthesized to amplify the full length of CAV genome (Table 1). The primers were synthesized by Sangon Biotech Co.,Ltd (Shanghai, China), and high fidelity DNA polymerase was used for amplification. The total DNA was extracted from the cultured cell supernatants of 33 CAV positive samples. PCR conditions were the same as described previously. Then, amplified products were sequenced and sequence assembly was carried out using the SeqMan program included in the DNASTAR software package (DNAStar Inc., Madison, WI, USA).

### Sequence Alignment and Phylogenetic Analysis

The genomic sequences and VP1 amino acid sequences of the isolates in this study and reference strains downloaded from GenBank were analyzed using DNASTAR software. The phylogenetic analysis was carried out by Neighbor-joining method by a bootstrap analysis of 1,000 replicates using MEGA 7.0 software program, and the criterion of CAV genotyping was referred to the previous studies (18, 21). The reference CAV strains downloaded from GenBank are listed in Supplementary Table 2.

### Recombination Analysis

Putative recombination events in the genomic sequences of the CAV isolates were identified using multiple methods in the Recombination Detection Program 4 (RDP v.4.97) software suite (23), and other detection methods, including RDP, GENECONV, BootScan, MaxChi, Chimera, SiScan, Phyl-Pro, LARD, and 3Seq, were also employed for comparison purpose. These putative recombinants events were further confirmed and visualized using SimPlot (24).

### Ethics Statements

The necropsy was approved by Shandong agricultural university animal care and use committee. Chickens suspected with CAV infection were sacrificed by well-trained operators and performed in accordance with the guidelines of the Committee on the Ethics of Animal of Shandong Agricultural University to protect animal welfare.

## Results

### Identification of CAV in Broilers, Shandong Province During 2020–2021

In order to investigate the CAV infection status of broilers in Shandong Province, 73 chicken samples suspected to be infected with CAV were collected during 2020–2021. The result of PCR detection revealed that 38 clinical samples (38/73, 52.05%) were CAV positive. Then, those 38 samples were homogenized and inoculated into MDCC-MSB1 cells for virus isolation. To investigate whether field CAV strain isolated, cellular supernatants were detected by PCR. In total, 33 strains of CAV were successfully isolated in the cell culture (33/38, 86.84%).

### Molecular Characteristic of Full-Length Genomic Sequence

**Figure 1:**
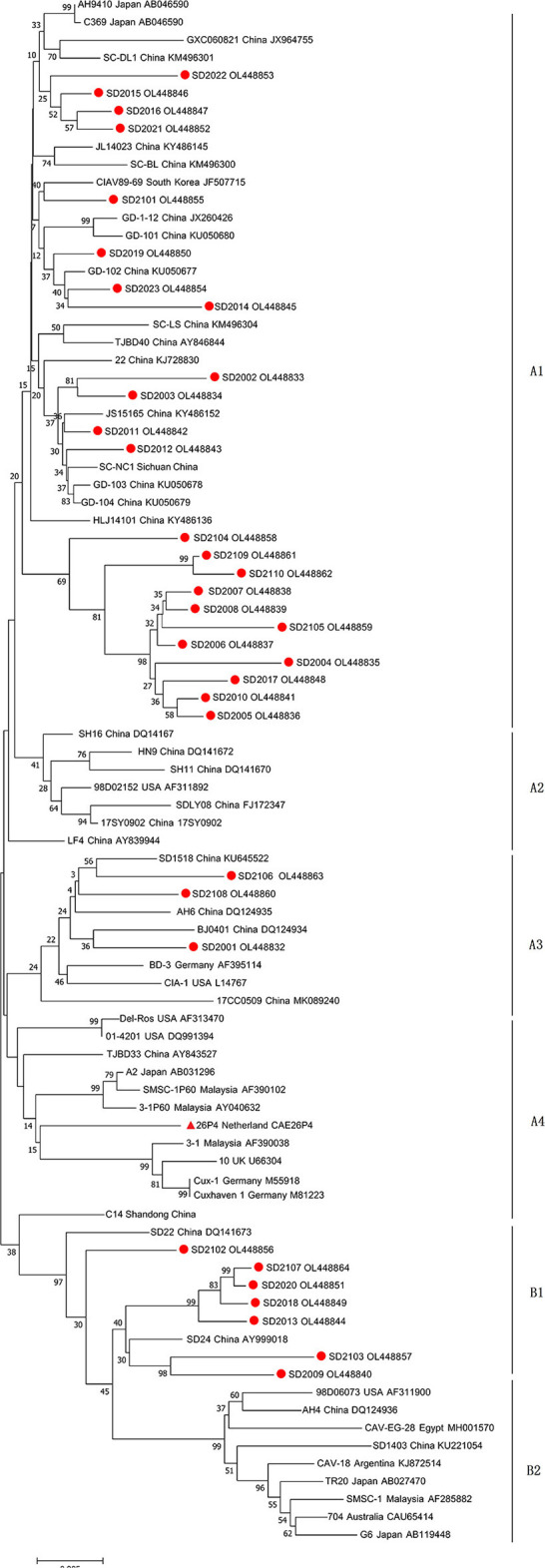
Phylogenetic tree of full-length genome sequences of 33 strains isolated in this study and reference CAV strains. The phylogenetic analysis was carried out by Neighbor-joining method by a bootstrap analysis of 1,000 replicates using MEGA 7.0 software program. The 33 strains isolated in this study were labeled with red circles, and attenuated vaccine 26P4 strain was labeled with red triangle.

Full-length genome sequences of 33 CAV strains isolated in this study were characterized and uploaded to GenBank database (Table 2). The complete genome length of all 33 CAV was 2,298 bp, encoding three overlapping open reading frames (ORFs), namely VP1, VP2, and VP3, and there was no insertion or deletion in the coding region. The genomic nucleotide sequences of 33 CAV strains had a 95.7–99.3% homology with each other and 95.3–99.3% homology with reference strains from GenBank. The homology between SD2107 and SD15 (NO. KX811526, isolated in Shandong, China, 2015) was the lowest (95.3%), while the homology between SD2012 and SC-NC1 (NO. KM496308, isolated in Sichuan, China, 2014) was the highest (99.3%). Then, phylogenetic analysis was performed using the complete genome sequences of 33 CAV strains and reference strains. As shown in Figure 1, all the CAV strains could be divided into group A and group B, in which group A was divided into four genotypes and group B was divided into two genotypes. Novel CAV strains isolated in this study were scattered across different branches, of which 23 strains were clustered in genotype A1, which was mainly composed of strains from China, Japan, and South Korea, and three strains were clustered in A3 genotype, which included CIA-1 (United States) and BD-3 (Germany). Interestingly, seven CAV strains isolated in this study were clustered in B1 genotype, along with few reference strains clustered in B1 genotype including SD22, SD24, and C14, which also isolated in Shandong province previously.

**Table 2:** Details of the 33 novel CAV genome sequences.

**No**.	**Accession No**.	**Strain name**	**Year**	**Biological sample**
1	OL448832	SD2001	2020	Bone marrow
2	OL448833	SD2002	2020	Bone marrow
3	OL448834	SD2003	2020	Spleen
4	OL448835	SD2004	2020	Liver
5	OL448836	SD2005	2020	Liver
6	OL448837	SD2006	2020	Bone marrow
7	OL448838	SD2007	2020	Bone marrow
8	OL448839	SD2008	2020	Liver
9	OL448840	SD2009	2020	Bone marrow
10	OL448841	SD2010	2020	Bone marrow
11	OL448842	SD2011	2020	Spleen
12	OL448843	SD2012	2020	Bone marrow
13	OL448844	SD2013	2020	Bone marrow
14	OL448845	SD2014	2020	Bone marrow
15	OL448846	SD2015	2020	Liver
16	OL448847	SD2016	2020	Spleen
17	OL448848	SD2017	2020	Liver
18	OL448849	SD2018	2020	Bone marrow
19	OL448850	SD2019	2020	Spleen
20	OL448851	SD2020	2020	Bone marrow
21	OL448852	SD2021	2020	Liver
22	OL448853	SD2022	2020	Bone marrow
23	OL448854	SD2023	2020	Spleen
24	OL448855	SD2101	2021	Bone marrow
25	OL448856	SD2102	2021	Liver
26	OL448857	SD2103	2021	Bone marrow
27	OL448858	SD2104	2021	Bone marrow
28	OL448859	SD2105	2021	Bone marrow
29	OL448863	SD2106	2021	Liver
30	OL448864	SD2107	2021	Liver
31	OL448860	SD2108	2021	Thymus
32	OL448861	SD2109	2021	Bone marrow
33	OL448862	SD2110	2021	Bone marrow

### Molecular Characterization of CAV VP1 Sequences

**Figure 2:**
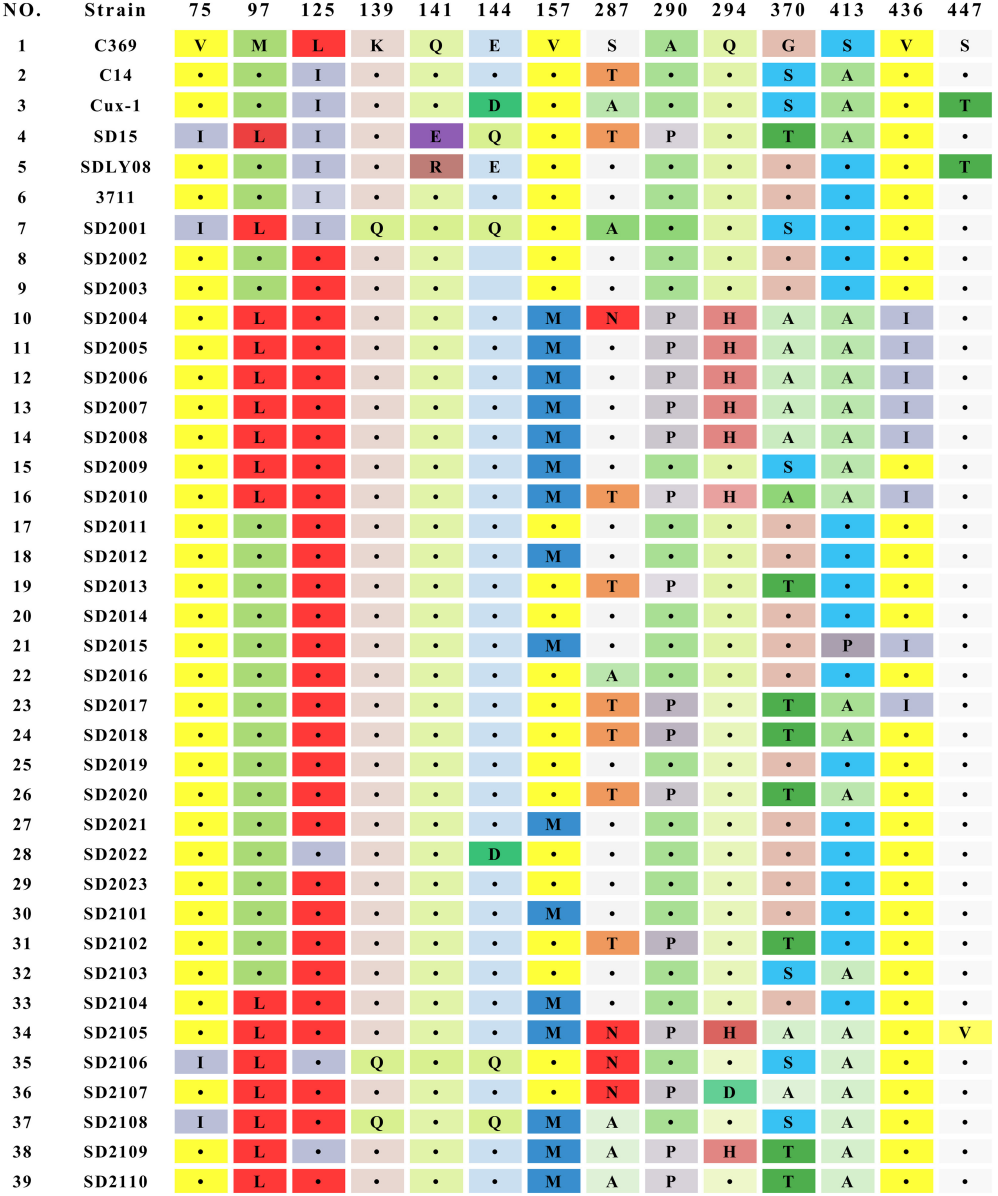
Amino acid substitutions observed in 33 CAV strains isolated in this study.

The result of sequence analysis showed that the length of VP1 gene of all CAV strains isolated in this study was 1,350 bp. The homology of the nucleotide sequences of 33 CAV strains ranged from 95.1 to 99.8%, showing a high degree of similarity. Analysis of the 450 amino acids of VP1 identified 35 amino acid substitutions, and mutations at important sites of VP1 were summarized in Figure 2. Among those important sites, positions 287 and 370 were the most variable sites, and different types of mutations were detected in 287 (S287A, S287N, and S287T) and 370 (G370S, G370A, and G370T). Previous studies had showed that positions of 75, 89, 125, 139, 141, 144, and 394 were associated with the viral replication and pathogenesis (5, 25, 26). In this study, all the 33 strains contained T89 and Q141, while valine (V) at positions 75 was replaced by isoleucine (I) in 3 strains (SD2001, SD2106, and SD2108), and leucine (L) at positions 125 was replaced by isoleucine (I) in 4 strains (SD2001, SD2022, SD2106, and SD2109), and glutamate (E) at positions 144 were replaced by glutamine (Q) in 3 strains (SD2001, SD2106, and SD2108). However, the simultaneous amino acid substitutions at the five positions were not observed among the 33 novel sequences. In addition, SD2001, SD2106, and SD2108 carried both Q139 and Q144, suggesting that these three strains might have relatively low rates of growth and spread efficiency. Lastly, all strains contained Q at positions 394, implying that all 33 strains might be highly pathogenic strains.

### Detection of Putative Recombination Events

**Figure 3:**
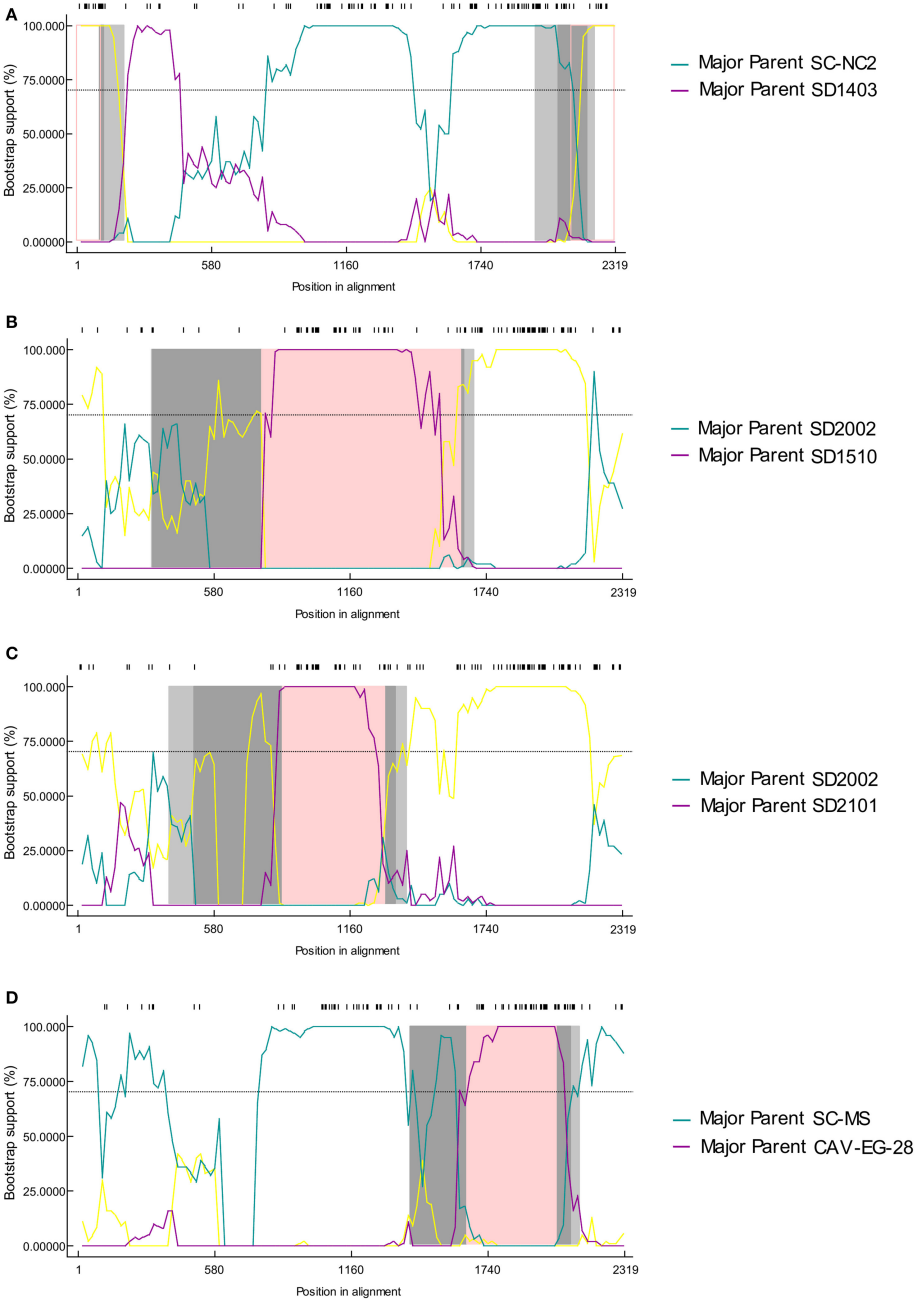
The bootscanning analysis of putative recombinants events in CIAV strain SD2014 **(A)**, SD2009 **(B)**, SD2103 **(C)**, and SD2102 **(D)**.

The possible genome recombination events were predicted using RDP4 software. When five of the nine methods in the RDP4 package are supported, it was determined that prediction of recombination events reliable. Four putative recombination events were identified, named SD2014, SD2009, SD2103, and SD2102 (Figure 3). For the strain SD2014, the breakpoints were detected to be approximately located at positions 2,128 and 108 (Table 3), which was from the major parent of SD1403 (isolated from Shandong, China) and the minor parent of SC-NC2 (isolated from Sichuan, China). The details of recombination sites and parents of another three strains were exhibited in Table 3.

**Table 3:** Putative recombination sites in CAV strains isolated in this study.

**Strain**	**Begin**	**End**	**Regions**	**Minor parent**	**Major parent**
SD2014	2,128	108	Non-coding	SD1403 (China)	SC-NC2 (China)
SD2009	784	1,636	VP1	SD1510 (China)	SD2002
SD2103	870	1,314	VP1	SD2101	SD2002
SD2102	1,653	2,037	VP1	CAV-EG-28 (Egypt)	SC-MS (China)

## Discussion

Since its first isolation in Japan in 1979 (10), CAV had become a ubiquitous pathogen in chicken farms worldwide. It could induce immunosuppression especially in young chickens, leading to the secondary bacterial, viral and fungal infection, which poses a serious economic threat to the poultry industry (2). CAV was mainly transmitted through horizontal contact or vertical transmission through eggs (27). In addition, the use of contaminated attenuated vaccine was also considered to be an important way of CAV transmission (28). CAV infection was first reported in China in 1996, and then spread to almost all provinces in China, which brought great threat to poultry production. Epidemiological investigations in recent years demonstrated that CAV is widely infected in Chinese chickens, including broilers, laying hens and Chinese indigenous breeds (17–21). However, there were no commercial CAV vaccines available in China at present.

In recent years, the clinical symptoms of suspected CAV infection such as growth retardation, thymus atrophy and yellow bone marrow of broilers in Shandong Province had been increased. In order to investigate the infection status and molecular characteristics of CAV strains in broilers in Shandong Province, 73 samples were collected during 2020–2021 for CAV detection and full-length genome amplification. Samples were taken from broilers of different breeds, ages, and generations, and the collection time was randomly distributed in each month. The results of PCR detection showed that 38 samples were positive for CAV. We speculated that other agents like reticuloendotheliosis virus (REV), avian leukosis virus (ALV), and infectious bursal disease virus (IBDV) contributed to the immunosuppressive symptoms in those CAV-negative samples, which needed further verification.

The molecular epidemiology of CAV had been previously described, and several genotypes of CAV had been determined by phylogenetic analysis (15, 16, 19, 29, 30). In this study, phylogenetic analysis using full-length CAV genomic sequences was performed according to previous studies. The results showed that most CAV strains (23/33) were clustered in A1 genotype with other Chinese isolates, of which 11 viruses clustered into the same evolutionary branch, indicating that CAV inheritance had a certain regional characteristic. It was worth noting that seven CAV strains clustered into B1 genotype with reference strains SD22, SD24, and C12. Genotype B group was mainly composed of reference strains from aboard, including United States, Argentina, Egypt, Australia, Malaysia, and Japan, while few Chinese isolates belonging to this genotype. Coincidentally, SD22, SD24, and C12 happened to be isolated in Shandong province previously. Therefore, we speculated that these strains might emerged through introduction of broiler breeders from aboard and then further evolved in Shandong province, China. This also reminded the importance of strengthening CAV detection in breeding chickens in the process of introduction.

CAV genome encoded three overlapping proteins: VP1, VP2, and VP3. VP1 had the highest mutation rate among the three viral proteins, which might be related to the selective pressure of host immune response. These variation sites were concentrated in some hypervariable regions of VP1, which had been proved to be related to the pathogenicity and replication of the virus (5, 25, 31). Amino acid position 394 was considered to be the main genetic determinant of virulence. In this study, all CAV strains carried Q394, indicating that all 33 CAV strains were highly pathogenic. In addition, positions at 75, 125, 141, and 144 in VP1 protein were also contributed to chicken pathogenicity. If amino acid substitutions occurred simultaneously at positions mentioned above, the pathogenicity of CAV would be attenuated (25). In this study, all CAV strains carried Q141, while three strains had V75I mutation, and four strains had E144D or E144Q mutation, indicating that most novel CAV strains had a strong virulence. Furthermore, it was worth noting that 48.5% (16 of 33 strains) of the isolates contained amino acid substitution at position 157 (V to M), which was higher than the substitution rate reported in previous studies, indicating that the evolutionary frequency of CAV might be accelerated (19). In contrast, no universal mutations were found in VP2 and VP3 proteins, indicating that VP2 and VP3 proteins were more conservative. This also suggested that VP2 and VP3 played fundamental functions in the infectious cycle of CAV.

Genetic recombination played an important role in virus evolution, and was essential in generating and maintaining genetic diversity of viruses. As a DNA virus, the genomic recombination of CAV had a low efficiency. However, previous studies had shown evidence of genetic recombination events (20, 21, 32). Consistent with these reports, reliable recombination events were also detected in this study. According to the published research, gene recombination of CAV could occur in coding region, non-coding region, and across coding region and non-coding region (18, 20). In this study, recombination events were detected in SD2014, SD2009, SD2103, and SD2102 strains, among which the recombination occurred in the non-coding region of SD2014, and VP1 gene of SD2009, SD2103, and SD2102. Interestingly, the recombination of SD2103 was occurred between SD2101 and SD2002. In terms of time, SD2103, SD2101 were isolated in the year 2021 while SD2002 was isolated in 2020. Geographically, all three viruses were isolated from the same area (Weifang City, Shandong Province). This demonstrated that mutual gene recombination could occur between different CAV strains in the same region, which increased the diversity of CAV genomes.

In conclusion, genomic characteristics of 33 CAV strains isolated in broilers in Shandong Province during 2020–2021 was reported in this study. This work provided more information for CAV epidemiology and genetic recombination, which will contribute to the further study of CAV genetic evolution.

## Data Availability Statement

The datasets presented in this study can be found in online repositories. The names of the repository/repositories and accession number(s) can be found in the article/Supplementary Material.

## Author Contributions

LG, YW, and PZ contributed to conception and design of the study. LL, YL, and MY organized the database. YW performed the statistical analysis and wrote the first draft of the manuscript. All authors contributed to manuscript revision, read, and approved the submitted version.

## Conflict of Interest

YL and LG were employed by the company Shandong Yisheng Livestock and Poultry Breeding Co., Ltd. The remaining authors declare that the research was conducted in the absence of any commercial or financial relationships that could be construed as a potential conflict of interest.

## Publisher's Note

All claims expressed in this article are solely those of the authors and do not necessarily represent those of their affiliated organizations, or those of the publisher, the editors and the reviewers. Any product that may be evaluated in this article, or claim that may be made by its manufacturer, is not guaranteed or endorsed by the publisher.
