# Supplementary material for: Genomic Characterization of Chicken Anemia Virus in Broilers in Shandong Province, China, 2020–2021
Source: Front Vet Sci. 2022 Mar 17;9:816860. doi: 10.3389/fvets.2022.816860 (PMC8968957; doi:10.3389/fvets.2022.816860)
Supplement: Supplementary file 1 [file Table_1.DOCX]

**Supplementary Table S1** Details of the samples collected and results of CAV detection.

| NO. | Date | Farm | Flock | Poultry company | Generation | Sample source | Age (weeks) | PCR Results | Virus isolate |
| --- | --- | --- | --- | --- | --- | --- | --- | --- | --- |
| 1 | 2020/1/23 | 1 | A | 1 | Parent | liver | 8 | Negative |  |
| 2 | 2020/1/25 |  | B | 1 | Parent | Bone marrow | 10 | Positive | SD2012 |
| 3 | 2020/1/27 | 2 | A | 1 | Parent | Bone marrow | 10 | Positive | SD2013 |
| 4 | 2020/4/18 |  | B | 1 | Parent | Liver | 7 | Positive | SD2017 |
| 5 | 2020/6/3 | 3 | A | 1 | Progenitor | Bone marrow | 14 | Positive | SD2022 |
| 6 | 2020/11/11 |  | B | 1 | Progenitor | spleen | 17 | Positive |  |
| 7 | 2020/12/2 | 4 | A | 1 | Parent | Bone marrow | 11 | Negative |  |
| 8 | 2020/12/27 | 5 | A | 1 | Parent | Bone marrow | 12 | Negative |  |
| 9 | 2020/12/27 |  | B | 1 | Parent | Bone marrow | 7 | Positive | SD2009 |
| 10 | 2020/12/8 | 6 | A | 1 | Parent | Bone marrow | 5 | Positive | SD2010 |
| 11 | 2021/1/4 | 7 | A | 1 | Parent | Bone marrow | 7 | Positive | SD2101 |
| 12 | 2021/4/8 | 8 | A | 1 | Parent | Bone marrow | 7 | Positive | SD2104 |
| 13 | 2021/6/23 | 9 | A | 1 | Broiler | Spleen | 2 | Negative |  |
| 14 | 2021/6/23 |  | B | 1 | Broiler | Bone marrow | 1 | Negative |  |
| 15 | 2021/10/18 |  | C | 1 | Broiler | Bone marrow | 2 | Positive | SD2109 |
| 16 | 2021/10/22 | 10 | A | 1 | Broiler | Bone marrow | 1 | Positive | SD2110 |
| 17 | 2020/5/18 | 11 | A | 2 | Parent | Bone marrow | 6 | Positive | SD2020 |
| 18 | 2020/10/2 |  | B | 2 | Parent | liver | 15 | Negative |  |
| 19 | 2021/3/21 | 12 | A | 2 | Parent | liver | 16 | Negative |  |
| 20 | 2021/8/10 |  | B | 2 | Parent | Bone marrow | 14 | Negative |  |
| 21 | 2020/3/18 | 13 | A | 3 | Parent | Liver | 7 | Positive | SD2015 |
| 22 | 2021/3/12 |  | B | 3 | Parent | Bone marrow | 11 | Negative |  |
| 23 | 2020/5/6 | 14 | A | 3 | Broiler | Liver | N.A. | Negative |  |
| 24 | 2021/3/7 |  | B | 3 | Broiler | Liver | N.A. | Positive | SD2102 |
| 25 | 2021/3/15 | 15 | A | 3 | Parent | Bone marrow | 11 | Positive | SD2007 |
| 26 | 2021/5/4 |  | B | 3 | Parent | Spleen | 13 | Negative |  |
| 27 | 2021/7/17 | 16 | A | 3 | Parent | Liver | 9 | Positive | SD2106 |
| 28 | 2020/9/18 | 17 | A | 4 | Parent | Liver | 7 | Positive | SD2005 |
| 29 | 2020/12/14 |  | B | 4 | Parent | Bone marrow | 8 | Positive | SD2001 |
| 30 | 2020/12/27 |  | C | 4 | Parent | Bone marrow | 8 | Positive | SD2002 |
| 31 | 2021/4/2 |  | D | 4 | Parent | Liver | 13 | Negative |  |
| 32 | 2021/6/30 |  | E | 4 | Parent | Bone marrow | 9 | Negative |  |
| 33 | 2021/7/4 |  | F | 4 | Parent | Bone marrow | 9 | Negative |  |
| 34 | 2021/7/17 | 18 | A | 4 | Parent | Bone marrow | 17 | Negative |  |
| 35 | 2021/8/9 |  | B | 4 | Parent | Thymus | 7 | Positive | SD2108 |
| 36 | 2021/9/6 |  | C | 4 | Parent | Bone marrow | N.A. | Negative |  |
| 37 | 2020/11/11 | 19 | A | 4 | Broiler | Spleen | N.A. | Positive | SD2003 |
| 38 | 2021/4/21 | 20 | A | 4 | Broiler | Bone marrow | N.A. | Positive | SD2105 |
| 39 | 2021/3/17 | 21 | A | 4 | Parent | Liver | 10 | Positive | SD2008 |
| 40 | 2021/7/12 |  | B | 4 | Parent | Bone marrow | 16 | Negative |  |
| 41 | 2021/8/9 |  | C | 4 | Parent | Bone marrow | 16 | Positive |  |
| 42 | 2020/4/13 | 22 | A | 5 | Broiler | Spleen | 1 | Positive | SD2016 |
| 43 | 2020/4/27 | 23 | B | 5 | Broiler | Bone marrow | 1 | Positive | SD2006 |
| 44 | 2020/5/12 | 24 | A | 5 | Broiler | Bone marrow | 1 | Negative |  |
| 45 | 2020/9/28 |  | B | 5 | Broiler | Bone marrow | 1 | Negative |  |
| 46 | 2020/6/10 | 25 | A | 6 | Broiler | Spleen | N.A. | Positive | SD2023 |
| 47 | 2020/7/19 | 26 | A | 7 | Broiler | Liver | N.A. | Negative |  |
| 48 | 2020/5/21 | 27 | A | 8 | Parent | Liver | 15 | Positive | SD2021 |
| 49 | 2020/8/23 |  | B | 8 | Parent | Bone marrow | 13 | Negative |  |
| 50 | 2020/10/6 |  | C | 8 | Parent | Bone marrow | 13 | Negative |  |
| 51 | 2020/10/13 | 28 | A | 9 | Broiler | Liver | 1 | Positive | SD2004 |
| 52 | 2020/10/22 | 29 | A | 10 | Broiler | Bone marrow | 1 | Negative |  |
| 53 | 2020/11/19 | 30 | A | 11 | Broiler | Bone marrow | 2 | Negative |  |
| 54 | 2021/3/17 | 31 | A | 12 | Broiler | Bone marrow | 1 | Positive | SD2103 |
| 55 | 2021/3/26 | 32 | A | 13 | Broiler | Liver | N.A. | Negative |  |
| 56 | 2021/5/6 | 33 | A | 14 | Parent | Bone marrow | 9 | Negative |  |
| 57 | 2021/5/8 |  | B | 14 | Parent | Thymus | 9 | Negative |  |
| 58 | 2021/6/28 | 34 | A | 15 | Broiler | Bone marrow | N.A. | Negative |  |
| 59 | 2021/10/22 |  | B | 15 | Broiler | Liver | N.A. | Positive |  |
| 60 | 2021/10/22 |  | C | 15 | Broiler | Liver | N.A. | Negative |  |
| 61 | 2021/7/19 | 35 | A | 16 | Broiler | Liver | 1 | Positive | SD2107 |
| 62 | 2021/8/25 | 36 | A | 17 | Broiler | Bone marrow | 2 | Negative |  |
| 63 | 2021/10/10 | 37 | A | 18 | Broiler | Bone marrow | 1 | Negative |  |
| 64 | 2020/3/5 | / | / | / | Backyard | Bone marrow | N.A. | Positive | SD2014 |
| 65 | 2020/4/20 | / | / | / | Backyard | Bone marrow | N.A. | Positive | SD2018 |
| 66 | 2020/4/21 | / | / | / | Backyard | Spleen | N.A. | Positive | SD2019 |
| 67 | 2020/4/28 | / | / | / | Backyard | Liver | N.A. | Negative |  |
| 68 | 2020/7/4 | / | / | / | Backyard | Liver | 1 | Negative |  |
| 69 | 2020/7/6 | / | / | / | Backyard | Spleen | 1 | Positive | SD2011 |
| 70 | 2021/3/11 | / | / | / | Backyard | Bone marrow | 1 | Negative |  |
| 71 | 2021/3/13 | / | / | / | Backyard | Bone marrow | N.A. | Positive |  |
| 72 | 2021/5/19 | / | / | / | Backyard | Bone marrow | 1 | Negative |  |
| 73 | 2021/10/20 | / | / | / | Backyard | Bone marrow | 1 | Positive |  |

Note: / not applicable, N.A. not available.
