# Supplementary material for: Genomic Characterization of Chicken Anemia Virus in Broilers in Shandong Province, China, 2020–2021
Source: Front Vet Sci. 2022 Mar 17;9:816860. doi: 10.3389/fvets.2022.816860 (PMC8968957; doi:10.3389/fvets.2022.816860)
Supplement: Supplementary file 2 [file Table_2.DOCX]

**Supplementary Table S2** Details of the reference strains used in this study.

| Strains | Time | Country | Accession NO. | Whole length |
| --- | --- | --- | --- | --- |
| CIA-1 | 1999 | USA | L14767 | 2298 bp |
| Del Ros | 2000 | USA | AF313470 | 2294 bp |
| 01-4201 | 2007 | USA | DQ991394 | 2298 bp |
| 98D02152 | 2010 | USA | AF311892 | 2298 bp |
| 98D02152 | 2010 | USA | AF311900 | 2298 bp |
| 704 | 1996 | Australia | U65414 | 2298 bp |
| 3-1 | 2003 | Malaysia | AF390038 | 2298 bp |
| 3-1 p60 | 2003 | Malaysia | AY390038 | 2298bp |
| SMSC-1 | 2003 | Malaysia | AF285882 | 2298 bp |
| SMSC-1P60 | 2003 | Malaysia | AF390102 | 2298 bp |
| Cux-1 | 2008 | Netherlands | M55918 | 2319 bp |
| CAE26P4 | 2007 | Netherlands | D10068 | 2298 bp |
| Cuxhaven 1 | 1992 | Germany | M81223 | 2298 bp |
| BD-3 | 2004 | Germany | AF395114 | 2298 bp |
| TR20 | 1999 | Japan | AB027470 | 2298 bp |
| G6 | 2009 | Japan | AB119448 | 2298 bp |
| A2 | 2000 | Japan | AB031296 | 2298 bp |
| C369 | 2001 | Japan | AB046590 | 2298 bp |
| AH9410 | 2001 | Japan | AB046589 | 2298 bp |
| 10 | 1997 | UK | CAU66304 | 2319 bp |
| CAV-EG-28 | 2018 | Egypt | MH001570 | 2298 bp |
| CAV-18 | 2014 | Argentina | KJ872514 | 2298 bp |
| CIAV89-69 | 2013 | South Korea | JF507715 | 2298 bp |
| C14 | 2004 | Shandong, China | EF176599 | 2298 bp |
| SD22 | 2005 | Shandong, China | DQ141673 | 2298 bp |
| SD24 | 2005 | Shandong, China | AY999018 | 2298 bp |
| SDLY08 | 2008 | Shandong, China | FJ172347 | 2298 bp |
| SD1403 | 2014 | Shandong, China | KU221054 | 2298 bp |
| SD15 | 2015 | Shandong, China | KX811526 | 2298 bp |
| SD1505 | 2015 | Shandong, China | KU645523 | 2298 bp |
| SD1507 | 2015 | Shandong, China | KU645507 | 2298 bp |
| SD1518 | 2015 | Shandong, China | KU645522 | 2298 bp |
| BJ0401 | 2004 | Beijing, China | DQ124934 | 2298 bp |
| TJBD40 | 2004 | Tianjin, China | AY846844 | 2298 bp |
| TJBD33 | 2005 | Tianjin, China | AY843527 | 2298 bp |
| HN9 | 2005 | Tianjin, China | DQ141672 | 2298 bp |
| LF4 | 2005 | Tianjin, China | AY839944 | 2298 bp |
| AH4 | 2005 | Anhui, China | DQ124936 | 2298 bp |
| AH6 | 2005 | Anhui, China | DQ124935 | 2298 bp |
| GXC060821 | 2012 | Guangxi, China | JX964755 | 2292 bp |
| SC-LS | 2014 | Sichuan, China | KM496304 | 2298 bp |
| SH11 | 2005 | Shanghai, China | DQ141670 | 2298 bp |
| SH16 | 2005 | Shanghai, China | DQ141671 | 2298 bp |
| GD-1-12 | 2012 | Guangdong, China | JX260426 | 2298 bp |
| GD-101 | 2013 | Guangdong, China | KU050680 | 2298 bp |
| GD-102 | 2013 | Guangdong, China | KU050677 | 2298 bp |
| GD-103 | 2013 | Guangdong, China | KU050678 | 2298 bp |
| GD-104 | 2013 | Guangdong, China | KU050679 | 2298 bp |
| SC-BL | 2014 | Sichuan, China | KM496300 | 2298 bp |
| SC-NC1 | 2014 | Sichuan, China | KM496308 | 2298 bp |
| 17CC0509 | 2017 | Jilin, China | MK089240 | 2298 bp |
| 17SY0902 | 2017 | Jilin, China | MK089243 | 2298 bp |
| JL14023 | 2014 | Jilin, China | KY486145 | 2298 bp |
| JS15165 | 2015 | JiangSu, China | KY486152 | 2298 bp |
| HLJ14101 | 2014 | Heilongjiang, China | KY486136 | 2298 bp |
| NX15140 | 2015 | Ningxia, China | KY486151 | 2298 bp |
| 22 | 2014 | Taiwan, China | KJ728830 | 2298 bp |
